# Shifts in doctor-patient communication between 1986 and 2002: a study of videotaped General Practice consultations with hypertension patients

**DOI:** 10.1186/1471-2296-7-62

**Published:** 2006-10-25

**Authors:** Jozien M Bensing, Fred Tromp, Sandra van Dulmen, Atie van den Brink-Muinen, William Verheul, François G Schellevis

**Affiliations:** 1 NIVEL, Netherlands Institute for Health Services Research, PO Box 1568, 3500 BN, Utrecht, The Netherlands; 2 Utrecht University, Department of Health Psychology, Heidelberglaan 1, 3584 CS, Utrecht, The Netherlands; 3 Utrecht Medical Center St. Radboud, Department of Postgraduate Training for General Practice, PO Box 9120, 6500 HB Nijmegen, The Netherlands; 4 VU, University Medical Centre, Department of General Practice, Van der Boechorststraat 7 1081 BT Amsterdam, The Netherlands

## Abstract

**Background:**

Departing from the hypotheses that over the past decades patients have become more active participants and physicians have become more task-oriented, this study tries to identify shifts in GP and patient communication patterns between 1986 and 2002.

**Methods:**

A repeated cross-sectional observation study was carried out in 1986 and 2002, using the same methodology. From two existing datasets of videotaped routine General Practice consultations, a selection was made of consultations with hypertension patients (102 in 1986; 108 in 2002). GP and patient communication was coded with RIAS (Roter Interaction Analysis System). The data were analysed, using multilevel techniques.

**Results:**

No gender or age differences were found between the patient groups in either study period. Contrary to expectations, patients were less active in recent consultations, talking less, asking fewer questions and showing less concerns or worries. GPs provided more medical information, but expressed also less often their concern about the patients' medical conditions. In addition, they were less involved in process-oriented behaviour and partnership building. Overall, these results suggest that consultations in 2002 were more task-oriented and businesslike than sixteen years earlier.

**Conclusion:**

The existence of a more equal relationship in General Practice, with patients as active and critical consumers, is not reflected in this sample of hypertension patients. The most important shift that could be observed over the years was a shift towards a more businesslike, task-oriented GP communication pattern, reflecting the recent emphasis on evidence-based medicine and protocolized care. The entrance of the computer in the consultation room could play a role. Some concerns may be raised about the effectiveness of modern medicine in helping patients to voice their worries.

## Background

Several authors have argued that there have been major changes in the doctor-patient relationship over the past decades; both from patients' and doctors' point of view [1-4]. There is, indeed, some evidence that changes in society and health care have resulted in real changes in what people expect from their doctors and in how doctors view patients [5]. Many patients want more information than they are given[5]. Many also say that they want to take an active part in decisions about their treatment, in the light of its chances of success and any side effects [6]. Concepts like 'patient empowerment', 'informed consent', 'shared decision making' and 'consumerism' have been introduced to label this transformation of the patient role from that of passive dependency to active autonomy [7]. According to the literature, the traditional paternalistic model is no longer the only, nor the preferred doctor-patient relationship model[8,9]. There is a wide consensus that a model based on a more equal doctor-patient relationship is both beneficial for patients and more in keeping with current ethical views [7].

However, there is also some evidence that reality in everyday practice is somewhat less 'advanced', for instance because many patients may not wish to be active participants in decision making[10]. The link between patient preferences for participation and actual participation is not very strong [1,6,7,11]. Patient preferences for information do not necessarily translate into information seeking behaviour; nor do patients who express preferences for some form of shared decision making necessarily act on these in the medical encounter [7]. In fact, a consistently very low level of patient question asking is shown in empirical studies throughout the years in a wide range of medical specialties [11-15]. In addition, the preference to participate in medical decision making does not seem to be universal. This preference appears to depend on age (with older people being less interested than younger people[10,16,17]), sex (men less interested than women [16,17]), education (patients with a lower educational level less interested than patients with higher education [16,17]), coping style (patients who are eager for information being more interested than others [18]) and severity of the medical problem (severely ill patients being less interested than healthy ones [19,20]).

Not only are patients said to have changed over the past years. Major changes have also taken place at the doctors' side; e.g. in medical education, organization of health care systems, and quality assurance programs. These changes may have resulted in better quality of care and maybe also in different types of doctors. On the one hand, there seems to be a tendency towards more affect-oriented 'patient-centred medicine' [21-24], which is one of the prevailing paradigms in modern medicine [21]. On the other hand, there has been a major shift towards a more rationalized, biomedically-oriented health care, based on protocols and guidelines (the paradigm of 'evidence based medicine') [25,26]. The scientific base of medicine has expanded tremendously, asking much of doctors in keeping up to date and drawing their attention to the technical side of medicine [27]. To which extent these two paradigms – patient-centred medicine and evidence-based medicine – have influenced the communication process itself cannot be stated with certainty, as empirical evidence about historical shifts in actual physician behaviour in medical encounters is largely absent.

The aim of our study is to compare patient and physician behaviour during routine consultations over a sixteen-year period in order to see how societal changes are reflected in the interpersonal interaction between general practitioners and their patients. Based on the hypotheses that over the last decades patients have become more active participants and physicians have become more task-oriented, this study tries to identify shifts in GP and patient communication patterns between 1986 and 2002. Our focus is on a homogeneous group of patients with a common health problem in general practice: hypertension. Hypertension was chosen, because its outcome depends on the quality of care as well as on the patient's active participation and self-management skills.

## Methods

### Setting and design

The study is a secondary analysis oftwo data sets containing observed primary care medical encounters collected in two distinct periods (1986 as against 2002) in the Netherlands. In both instances, a sample of approximately 100 consultations with hypertension patients were drawn from larger data sets and subjected to further analysis. The first wave consists of 102 visits, recorded by 27 general practitioners, drawn from a body of 1569 videotaped consecutive consultations with 'real-life' patients [28-30]. From this larger dataset all patients who consulted their GPs for hypertension were selected. The second wave is comprised of 108 visits, recorded by 108 GPs, which were chosen by selecting every first patient with hypertension per GP from a larger dataset (n = 2784) [31] of videotaped visits in general practice (one visit per GP).

### Sample

#### Patients

No differences were found in age, gender or (by selection) primary health problem between the two study samples. The patients' mean age was 57.7 (sd 14.95) and 61.4 (sd 14.66) years, respectively (n.s.) and 66% versus 63% of the sample was female (n.s.). In both samples the vast majority of the consultations were repeat visits.

There were no data available on patients' SES. However, in the Dutch health care system all patients are registered with a general practitioner and there are no financial barriers for patients consulting their general practitioners. Few Dutch patients seek routine care outside of the formal system.

#### Physicians

In both samples, all physicians were specialized in general practice. The majority (92 % versus 94 %) had more than 5 years experience. In the first wave, all of the physicians were white males, 43% were in solo practice, the rest in group practice or in multidisciplinary health centres. In the second wave, three out of four physicians (74 %) were white males and 35 % were in solo practice. Dutch GPs have fixed lists of patients for whom they are the first contact for all health problems and hold a gatekeeper position to specialized care. Routine care for hypertension patients is delivered in General practice

### Coding procedures and measures

The total *visit length *and the duration of the *physical examination *were stopwatch-timed in both datasets. *Communication patterns *were rated using the Roter Interaction Analysis System (RIAS), a widely-used international observation system with proven validity and reliability[13,29,32-34]. In RIAS every doctor and patient utterance is coded in mutually exclusive categories (see table 1 for examples). According to the RIAS-manual[35,36], "utterances" are defined as the smallest distinguishable speech segment to which a classification may be assigned. The unit may vary in length from a single word to a lengthy sentence. Talk that did not fit any of the categories ('other talk', including unintelligible talk) was included in the total of patient and doctor utterances. RIAS-categories were aggregated into bigger and meaningful categories, based on factor analysis and consistency with previous publications [29,30].

**Table 1:** Frequencies of communication categories per consultation by GPs and hypertensive patients between 1986 and 2002.

		**1986 (n = 102)**		**2002 (n = 108)**		
*Category*	*Example*	*e*^*μ*^	*95% CI*	*e*^*μ*^	*95% CI*	*P-value*

All GP talk		129	119.7–139.7	119	113.4–124.1	0.26
Task-oriented talk						
biomedical questions	"Did you feel dizzy lately?"	9.1	8.3–9.9	7.1	6.6–7.6	0.05
biomedical info & counselling	"Your blood pressure went down."	26.7	24.5–29.1	34.7	32.5–37.0	0.02
psychosocial questions	"Are you anxious about this?"	2.9	2.4–3.5	3.7	3.4–4.1	0.18
psychosocial info & counselling	"You need to get out and meet more people."	6.7	5.5–8.0	6.2	5.4–7.1	0.38
Affect-oriented talk						
social talk	"Your daughter is in 2nd grade now, isn't she?"	9.3	8.4–10.3	10.5	9.6–11.6	0.27
concern/optimism	"I'm really worried about your blood pressure."	5.5	5.0–5.9	1.4	1.2–1.6	< 0.001
rapport building, verbal attentiveness	"I can understand that this is distressing for you."	37.8	33.4–42.8	37.2	35.0–39.6	0.40
Process-oriented talk						
instructions, directions	"Now I'm going to take your blood pressure."	16.5	14.9–18.2	10.3	9.6–11.0	< 0.001
partnership building, dialogue seeking	"If I understand correctly,..."	4.3	3.7–4.9	1.5	1.3–1.7	< 0.001
disagreements	"No, you really should take the pills every day."	1.0	0.8–1.2	0.3	0.2–0.4	< 0.001
						
All patient talk		139	127.7–151.8	109	103.6–114.9	0.02
Task-oriented talk						
biomedical questions	"Can this be a side-effect of the medicines I take?"	6.0	5.4–6.5	4.3	4.0–4.7	0.01
biomedical info	"I don't have those headaches anymore."	36.8	33.6–40.4	33.1	30.9–35.5	0.27
psychosocial questions	"How does stress influence my blood pressure?"	0.5	0.4–0.7	0.5	0.4–0.6	0.40
psychosocial info	"I have been worrying about losing my job"	24.8	21.1–29.1	21.4	19.1–24.0	0.31
Affect-oriented talk						
social talk	"How were your holidays?"	10	8.6–11.7	11.8	10.5–13.2	0.28
concern/optimism	"These results got me pretty scared!"	12.3	11.2–13.4	1.0	0.8–1.2	< 0.001
rapport building, verbal attentiveness	"This must be a tough job."	24.9	22.6–27.4	29.4	27.8–31.1	0.13
Process-oriented talk						
Instructions, directions	"I want to talk about the consequences of this."	5.1	4.2–6.0	0.9	0.8–1.2	< 0.001
partnership building, dialogue seeking	"Let me see if I got this right,..."	3.2	2.9–3.5	0.4	0.4–0.5	< 0.001
disagreements	"No, I don't think the pills do much good"	1.0	0.8–1.2	0.4	0.3–0.5	0.02

e^μ ^is the frequency as estimated by the poisson regression model

When P-values are smaller than 0.05, differences are considered significant.

Although different observers coded the two samples, all coders had been extensively trained according to the same training protocol using the manual and material provided by the original author of RIAS[14]. For the first cohort the RIAS-manual of 1987 [35]was used. For the second cohort the 1993 update of this manual was used, which showed no relevant changes compared to the 1987 manual [36]. The first author supervised the training as well as the coding in both samples. In both samples the inter-observer reliability of RIAS categories was assessed as satisfactory to very good with Pearson's r ranging from 0.72 to 0.99.

### Statistical analysis

We used the software package MlwiN 2.01 [37] to analyse the data. Frequencies and 95% confidence intervals of utterances in RIAS categories and the duration of the visit and physical examination were estimated using a Poisson model with extra Poisson variation to account for over-dispersion. The models were fitted using second order Penalized quasi-likelihood (PQL) estimation. The Poisson distribution was used, because the distributions of the outcome variables were highly skewed and also to attain meaningful (positive) estimates for the confidence intervals of the count data. All models were adjusted for clustering at the GP level by using a random intercept constant at GP level. Since clustering was present only in the 1986 sample, the random intercept was only applied for these consultations by means of a dummy variable, which was 1 for the 1986 sample and 0 for the 2002 sample. When a model failed to converge, first order PQL estimation was used, which yields slightly less accurate confidence intervals. This was done for GP Psychosocial questions and GP Psychosocial information. We used z tests based on the Poisson model to establish if the estimates of the outcome variables differed between the two groups.

### Ethical approval

This study was carried out in accordance with Dutch privacy legislation. All participating doctors and patients gave their informed consent.

## Results

Visit length was slightly, but not significantly longer in recent consultations (9.0 minutes in 1986 versus 10.0 minutes in 2002; P = 0.18); no differences emerged in time spent on physical examination (2.2 minutes in 1986 versus 2.0 minutes in 2002; P = 0.23).

The differences in physician and patient communication behaviour are presented in Table 1. The amount of talk by doctors did not significantly differ between the 1986 and the 2002 sample (P = 0.26), but patients did talk less in recent consultations (139 versus 109 utterances, P = 0.02). Compared with the 1986 wave, patients in the 2002 wave asked fewer medical questions, showed less concerns or worries and had fewer process-oriented interventions, like asking for clarification or partnership building. Doctors in 2002 also asked fewer biomedical questions, but they provided significantly more medical information as compared to 1986. Just like the patients, GPs had fewer process-oriented interventions in 2002 and expressed less often their concern about the patients' medical condition.

In order to find an explanation for the unexpected decrease in the amount of patient talk in recent consultations that could not be explained by consultation length or duration of the physical examination, a closer inspection of the videotaped consultation was made. The main difference was found in silences, due to physicians' computerized record keeping. In 1986 none of the physicians had a computer on their desks; by 2002, all of them did. On average nearly 2 minutes were spent on computerized administrative work (mean = 112 seconds; sd = 92.5 seconds; scores ranging from 0 to 525 seconds).

## Discussion

Information giving is an important element in the quality of care: patients need information in order to understand their condition[38]; to acquire a feeling of control, necessary for successful self-management[38]; and to participate in medical decision-making [39,40]. In 2002 the GPs showed a greater amount of information giving Nevertheless, our data also show a shadow-side in physician's changed behaviour. The 2002 physicians were less engaged in partnership building, for instance by asking for patients' opinions, asking for clarification of patients' words, or giving explicit structure to the consultation. They also expressed less often their concern for the patients' medical condition. All in all, the general practitioners from our 2002-sample seem to be more task-oriented than the GPs from the 1986-wave who asked more questions and sought more interaction with their patients.

These findings are in line with the results on patients' side of the communication. All in all, the hypertension patients from the 2002-wave made a substantially smaller contribution to the consultation than their 1986-counterparts, mainly in the process-oriented domain They asked fewer biomedical questions and engaged less in partnership building with the general practitioner, for instance by asking for clarification. Moreover they talked substantially less about what was bothering them. These findings are contrary to expectations, based on the theoretical literature about patient autonomy. In recent publications, it has been argued that patient participation is important for hypertension patients, because, once on medication, the hypertension patient will largely have to manage him/herself [39,41]. Following the literature on patient autonomy, a rise in patient question-asking and process-oriented interventions would have been expected, as modern patients are said to want more information and to be more actively engaged in the medical consultation. For this controversy several explanations are possible. In the first place, it can be argued that patients who visit their general practitioner with hypertension are usually older and therefore don't fit into the model of the modern autonomous patient. Older people are indeed known to have less preference for shared decision making than younger patients[10,16]. This could be either an age-effect or a cohort-effect[10]. However, since no age differences existed between the two samples and a cohort effect would indeed have led to higher patient participation in the 2002 group, age cannot be an explanation for diminished patient participation in our recent sample. Theoretically, it is possible that general practitioners in the 2002 sample were so comprehensive in their information giving that patients had no remaining questions. However, this is quite unlikely. It is at odds with the evidence that doctors consistently underestimate patient's desire for information and that they are not good at eliciting patient preferences[10]. In a recent Dutch study, one third of all general practice patients left the consultation room with unasked questions, 30 % blaming lack of time, 19 % unclear information from the general practitioner and 21 % reported to be too stressed to ask all questions [42]. In a recent qualitative study, only four out of 35 patients said all they wanted to say during the consultation[43]. Maybe today, hypertension patients are monitored more extensively than sixteen years ago and are called in to visit the general practitioner more often for a bodily check-up including blood pressure taking. It is, however, questionable if an increase in the number of visits actually decreases the number of questions asked by patients per visit. Perhaps the most plausible explanation for the low patient participation found in our study is that hypertension patients simply have not turned into active, independent and emancipated consumers, as we are led to believe they would; at least, not during medical consultations. This does not mean that these patients are not autonomous people outside the consultation room. Indeed outside the consulting room patients seem full of ideas on their medical condition and opinions of medical treatment [44].

While this could explain why patient contribution in the medical consultation is low, it does *not *explain why patients' level of activity is now lower than sixteen years ago. Maybe shifts in physician's behaviour could provide an explanation. Most authors agree that physicians set the agenda for the consultation, and patients follow [7]. Doctors need to create opportunities for patients to feel comfortable in expressing their real worries[45]. 'Being able to talk' has been found to be the most important element in a consultation [10,45], and it is up to doctors to encourage patients to reveal more of what's on their minds[45]. It has been demonstrated that general practitioners' style of listening to the patient will influence what the patient says[46]. Now, the main shift in doctor communication behaviour that we found in this study is a shift from process-oriented towards task-oriented communication – mainly biomedical information giving. Information-giving fits within the first need of what George Engel[38] has called: 'patients double need', i.e.: 'the need to know and understand', a cognitive need related to patients' own task-oriented coping efforts, such as involvement in medical decision making. Information giving clearly does not help to fulfil patients' second need: 'the need to be recognized and understood', an affective need related to patients' emotional coping efforts, such as revealing worries and concerns [45]. Both elements, i.e. supporting patients in shared-decision making for instance by providing as much information as patients need versus facilitating patients to reveal complex agendas, are central in the concept of patient-centeredness [22-24]. Our study shows that Dutch GPs have increased their information giving, but might have lost some of their former capacity to let patients talk along the way. A last possible explanation has to do with the entrance of the computer into the consultation room. None of the GPs from the first cohort were using a computer, while all of the GPs our more recent sample did. And as we have seen, they spent a considerable of amount on computerized record-keeping. Whereas patients used to continue talking when the physician wrote down his findings on paper in the first wave, they tended to remain silent when the physician was using his computer in the second wave. Moreover, it could well be that the computer further contributed to the already more businesslike atmosphere of the recent consultations. Margalit el al[47] found that physician's gazing at the monitor was inversely related to physician engagement in psychosocial questioning and emotional responsiveness as well as to patient socioemotional and psychosocial exchange during the visit and that it diminished the dialogue between physician and patient. It could be that GPs are not yet completely used to computerized record-keeping, and need some time to adjust to this new 'third party' in the consultation room. However, it seems that both research and education should pay more attention to the influence of the computer on the course of medical consultations in order to minimize the disruptive and maximize the beneficial effects of this new companion of doctor and patient.

### Strengths and weaknesses of the study

A strong point of this study is that communication patterns of both physicians and patients could be compared over a fifteen year period, using exactly the same methodology: observation of videotaped, real-life General Practice consultations with hypertension patients. The patient characteristics of both samples were comparable, thus adding to the plausibility that the differences in results between the two time periods are indeed a reflection of shifts in communication. Another strong point is that all statistical analyses have been guided by clear hypotheses, which were articulated before this study started.

The study also has some weaknesses. In the first place, different coders coded the two samples. However, all coders had been extensively trained according to the same training protocol, and were supervised by the first author. The 1987-manual was used for coding the first sample, the 1993-update for coding the second sample. The definitions of the codes in the 1993 update were not different in content from those in the 1987 manual, only more explanation and examples had been added to the 1993 manual. The complete text of both manuals can be requested from the authors. The high reliability scores of RIAS in both samples, similarly as found in earlier international RIAS studies, give us additional confidence that it is not very likely that the differences in results should be ascribed to the different coders.

Another potential weakness of the study is the fact that the two samples differed in the number of patients per GP. In the first study, there were several hypertension patients per GP, while in the second study only one hypertension patient per GP was included. As a consequence, consultations in the first sample were clustered around doctors, which asked for a multilevel approach, while in the second cohort no multilevel approach was needed. After consultation of a statistical expert, we decided to do a multilevel analysis, that controlled for the clustering in only in the first cohort. It is important to note that the statistical approach that we used yielded almost identical results than the conventional single-level approach. Seen the data structure and the distribution of de dependent variables, we think that the multi-level Poisson model provides the most accurate estimates of the differences in communication in both periods.

## Conclusion

In conclusion, Dutch GP's had a more task-oriented communication style in 2002 compared to 1986. It appears that patients did not become more active participants in the consultation: patients talked less, asked fewer biomedical question and showed fewer concerns. Contrary to our expectations, a shift to a more egalitarian relationship in General Practice was not found.

## Competing interests

J Bensing, F Tromp, S van Dulmen, A van den Brink-Muinen W Verheul and F Schellevis all declare that they have no conflict of interest.

## Contributors

JMB and FT ran the project from day to day. WV did the analyses for the paper. JMB initiated and planned the project, assisted by SvD, AvdBM and FGS. JMB wrote the initial draft of the paper and all authors contributed to the final version. All authors had full access to all the data in the study and all authors have seen and approved the final version.

## Pre-publication history

The pre-publication history for this paper can be accessed here:


